# The contemporary pulmonary artery catheter. Part 1: placement and waveform analysis

**DOI:** 10.1007/s10877-021-00662-8

**Published:** 2021-02-10

**Authors:** I. T. Bootsma, E. C. Boerma, F. de Lange, T. W. L. Scheeren

**Affiliations:** 1 Department of Intensive Care, Medical Center Leeuwarden, Henri Dunantweg 2, P.O. Box 888. 8901, Leeuwarden, The Netherlands; 2 Department of Anesthesiology, University of Groningen, University Medical Center Groningen, Groningen, The Netherlands

**Keywords:** Hemodynamic monitoring, Pulmonary artery catheter, Waveform characteristics, Pulmonary artery pressure

## Abstract

Nowadays, the classical pulmonary artery catheter (PAC) has an almost 50-year-old history of its clinical use for hemodynamic monitoring. In recent years, the PAC evolved from a device that enabled intermittent cardiac output measurements in combination with static pressures to a monitoring tool that provides continuous data on cardiac output, oxygen supply and-demand balance, as well as right ventricular (RV) performance. In this review, which consists of two parts, we will introduce the difference between intermittent pulmonary artery thermodilution using cold bolus injections, and the contemporary PAC enabling continuous measurements by using a thermal filament which at random heats up the blood. In this first part, the insertion techniques, interpretation of waveforms of the PAC, the interaction of waveforms with the respiratory cycle and airway pressure as well as pitfalls in waveform analysis are discussed. The second part will cover the measurements of the contemporary PAC including measurement of continuous cardiac output, RV ejection fraction, end-diastolic volume index, and mixed venous oxygen saturation. Limitations of all of these measurements will be highlighted there as well. We conclude that thorough understanding of measurements obtained from the PAC are the first step in successful application of the PAC in daily clinical practice.

## Introduction

In 1970 the floating pulmonary artery catheter (PAC) was introduced by Swan and Ganz [1]. The underlying objective of the two physicians was to apply physiologic principles to the understanding of the circulatory abnormalities characterizing an illness in an individual patient, and to provide a rational basis for selection of therapy with objective, quantitative assessment of patient response [1]. The principal stimulus for the development of the PAC was the aim to study and improve the care of acutely ill patients in whom fluoroscopy was not readily available or who were not in a condition to be readily moved to a diagnostic facility [1]. Despite these noble intentions, over time the PAC has predominantly become a topic of debate concerning safety, indication and clinical utility, with the main focus on the potential of the PAC to improve clinical patient outcome [2–4]. Fuelled by large randomized controlled trials (RCT) that failed to demonstrate any outcome benefit in relation to PAC-use in a large variety of disease states, the verdict on general application in the clinical setting has become predominantly negative [5–8]. In spite of this the use of PAC’s is still widespread, especially in the fields of cardiology and cardiac surgery [9, 10]. This seeming controversy may be due to the understanding of clinicians on the potential limitations of PAC oriented RCT’s, including patient selection, timing and the general absence of a protocolized strategy based on PAC-derived variables [5, 11–14]. However, the most probable explanation might be that clinicians from all over the world value the fundamental understanding of physiological principles in the management of complex disease states [15, 16]. In this respect it remains key to acknowledge that adequate interpretation of PAC-derived data requires both skills and knowledge about the correct use of the device, as well as of its pitfalls. The classical PAC evolved from a device that enabled intermittent cardiac output measurements in combination with static pressures to a contemporary PAC, which in turn provided continuous data on cardiac output (CCO-PAC), oxygen supply and demand balance, as well as right ventricular (RV) performance. This CCO-PAC, further mentioned as PAC, is a 7.5 F continuous cardiac output/mixed venous oxygen saturation [SvO_2_]/continuous end diastolic volume [CEDV]-pulmonary artery catheter (model 774F75; Edwards Lifesciences, Irvine, CA, USA).

The additional information that comes from these technological innovations is specifically included in this review. This narrative review reflects a concise overview of the available knowledge. In the first part of this review, we will discuss catheter placement, waveform characteristics, and pitfalls. In the second part we will describe technical features, clinical applications, limitations, and complications of this contemporary PAC.

## Placement of the pulmonary artery catheter

The PAC is introduced via a dedicated sheath during a sterile procedure using the Seldinger technique. Ultrasound guidance during catheter placement is highly recommended [17, 18]. Placement of the sheath can be into either one of the internal jugular veins (IJV), the subclavian veins, or the femoral veins. The right IJV is the favoured site for sheath placement since it provides the most direct route towards the right ventricle. Although subclavian access is associated with fewer infectious complications compared to femoral or jugular access, bleeding complications may have more serious consequences and anatomical location and vessel size vary considerably [19–21]. Furthermore, ultrasound visualization of the subclavian vein is technically demanding, due to interference by the collarbone [22]. After successful placement of the introducer, the PAC can be inserted through the sheath. The PAC is 110 cm in length, marked at 10 cm intervals, and has at least 2 channels. The distal channel at the tip of the catheter allows for transducing pulmonary artery pressure (PAP) and SvO_2_ sampling, while the proximal channel is used for measuring central venous pressure (CVP) and central venous saturation (ScvO_2_) sampling. A balloon is located just below the tip of the catheter, in which 1.5 ml of air can be inflated once the PAC is inserted beyond the sheath (at least 20 cm). Before placement, in vitro calibration of the SvO_2_ fiberoptic should be performed using a photodetector before removal of the catheter from the package. After calibration, the catheter can be connected to the monitor and transducer. Subsequently, both the proximal and distal channel should be flushed and filled with fluid. In case in vitro calibration of the fiberoptic is not performed, in vivo calibration may be performed after correct placement of the catheter by drawing a blood sample from the distal channel and analysing this sample for SvO_2_. It is of note that this calibration process is only applicable for the contemporary PAC and not for the older PAC, which obtains cardiac output from intermittent thermodilution. A detailed description on SvO_2_ can be found in part two of the review.

**Fig. 1:**
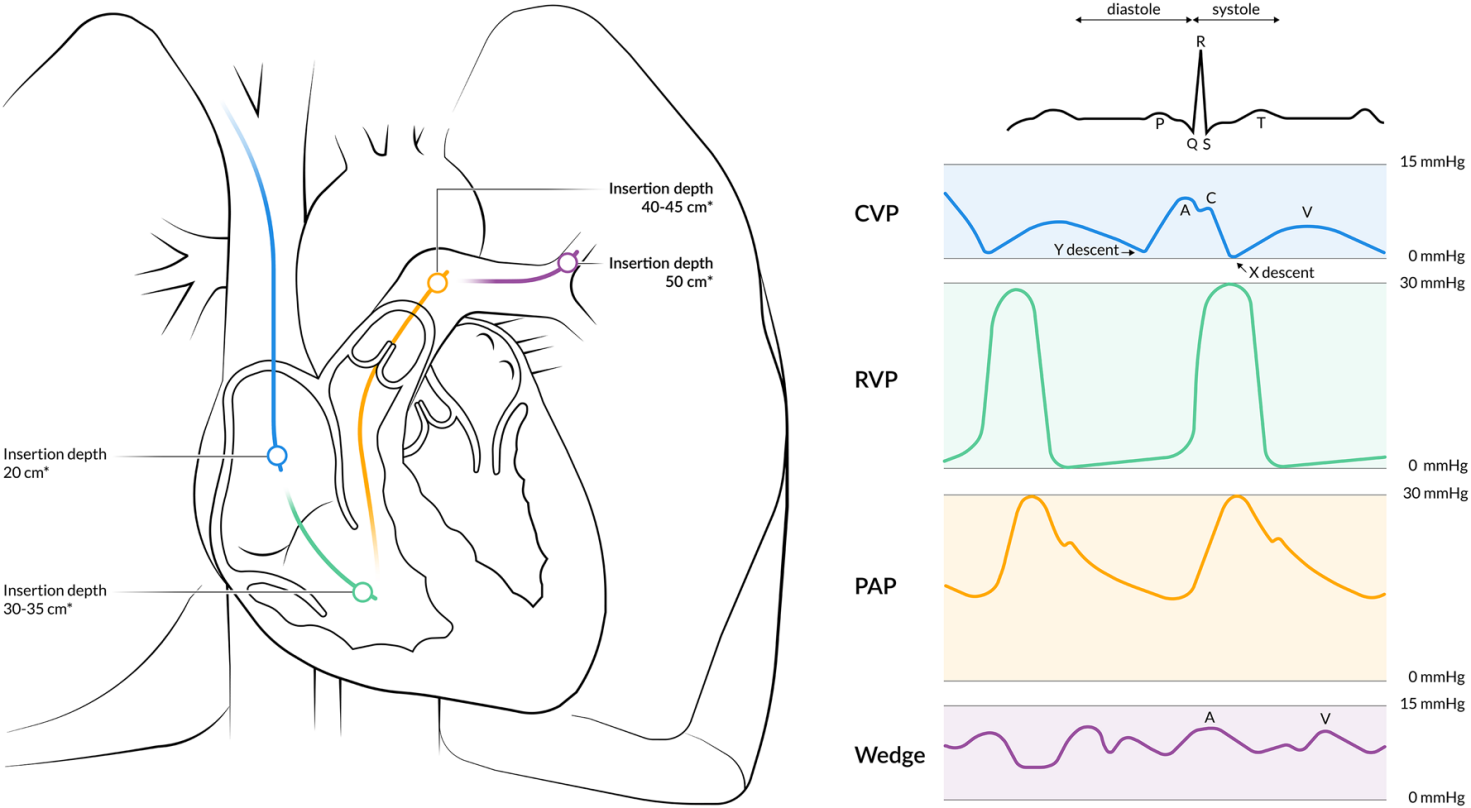
Placement of the PAC guided by the characteristics of normal vascular pressures and waveforms . *For placement in the left internal jugular vein or left subclavian vein one should add 5 cm to each of the landmarks . *CVP *central venous pressure*, PAC *pulmonary artery catheter,* PAP *pulmonary artery pressure,* RVP *right ventricular pressure

Placement of the PAC is guided by the characteristics of vascular pressures and waveforms (Fig. 1). In order to facilitate this, the distal lumen of the catheter should be attached to a pressure transducer. Despite individual variety, specific landmarks are well-related to insertion length, depending on the puncture site. After introduction via the right IJV or the right/left subclavian vein, the right atrium should be reached at approximately 20 cm insertion depth; the right ventricle at 30–35 cm, the pulmonary artery at 40–45 cm, and the wedge position at 50 cm (Fig. 1) [23]. For the left IJV one should add 5 cm to each of the previously mentioned landmarks. However, in populations with shorter statures, the insertion length is usually less deep [24]. In case of heart failure with dilatation of the RV, or in tall patients, an insertion length of greater than 50 cm might be necessary. When removing the PAC from its packaging, it has a natural curvature which should be pointed towards the heart. Counter clockwise rotation during insertion with an inflated balloon increases the odds of entering the right atrium and passing the tricuspid valve [25]. When the RV waveform does not appear after 40–45 cm of insertion, or if the PAP waveform does not appear after 50–55 cm, the balloon should be deflated and the catheter should be withdrawn until 20 cm with subsequent repetition of the procedure. To facilitate successful placement of the PAC, positioning the patient head-down will aid flotation past the tricuspid valve. In order to facilitate the passage through the pulmonary valve, positioning the operation table or ICU bed with head up (15°–20°) and rotated to the right may be helpful [23, 26]. Most catheters float easily toward the right pulmonary artery catheter. In order to selectively catheterize the left pulmonary artery, the patient should be positioned with the right side down. In the setting of low cardiac output, deep inspiration in non-intubated, spontaneously breathing patients will increase right ventricular output transiently and therefore may facilitate catheter flotation [23]. After correct placement, in vivo calibration of the fiberoptic should be performed.

### Zeroing

Zeroing and leveling of the catheter are a prerequisites to obtaining accurate measurements, and both have revealed to be susceptible to error [27]. Opening the stopcock to ambient air, the hemodynamic monitoring system will be exposed to atmospheric pressure. After pressing ‘zeroing’ on the monitor and confirming the calibration, the transducer stopcock can be turned back into its original position. The atmospheric pressure is now the zero-reference point. From there on, only (variations in) pressures which exist inside the heart chamber or blood vessel will be measured, as long as the position of the pressure transducer remains the same [28].

### Leveling

The main goal of leveling the external transducer is to eliminate additional hydrostatic pressure from the fluid column. This hydrostatic pressure is proportional to the height of the fluid column. The level of the transducer should be even with the top of the fluid column in the chamber or vessel in which the pressure is to be measured [29]. The correct position in supine patients is the phlebostatic axis, which is about 5 cm below the sternal angle [30]. When patients are in prone or sitting position reference levels might be different [31]. In case the transducer is placed above the phlebostatic axis, the pressure will be underestimated. Vice versa, the measured pressure will be erroneously high in case the transducer is placed below the phlebostatic axis.

### Waveforms of the pulmonary artery catheter

#### Central venous/right atrial pressure waveform

Initially, the PAC is passed through the introducer sheath until it reaches the IJV, the superior vena cava, and the right atrium. Reaching this point, the monitor will depict either a CVP or right atrial pressure waveform, which are considered to be identical. A normal CVP waveform consists of 5 phases; three peaks (a-wave: atrial contraction; c-wave: isovolumic ventricular contraction, tricuspid motion toward right atrium; v-wave: systolic filling of the atrium), and two troughs (x: atrial relaxation; y: early ventricular filling). Identification of CVP waveform components is facilitated by aligning the pressure waveform with the ECG trace. The a-wave follows the ECG P-wave, the c-wave always follows the ECG R-wave and the v-wave follows the ECG T-wave [32]. CVP should be measured at the base of the c-wave, just after the R-wave of the ECG, because this represents the final pressure in the ventricle before onset of the systole. If the c-wave is not identified and the patient has sinus rhythm, the base of the a-wave can be used. A normal CVP range in healthy, spontaneously breathing humans in the supine position is between 0 and 10 mmHg (Fig. 1) [33].

#### Right ventricular pressure waveform

When advancing the PAC with an inflated balloon through the tricuspid valve, RV pressures will be recorded. The major difference with the CVP characteristics is a marked increase in systolic RV pressure. A normal RV-waveform is characterized by a steep, rapid systolic slope. Due to the substantial compliance of the normal RV, the diastolic slope is typically horizontal [34, 35]. Diastolic pressure in the RV of a healthy individual is almost equal to zero. End diastolic pressure is measured right before the R-wave on the ECG, before the beginning of the systolic upslope [34]. Normal systolic pressure of the RV ranges between 15 and 28 mmHg (Fig. 1).

#### Pulmonary artery pressure waveform

**Fig. 2:**
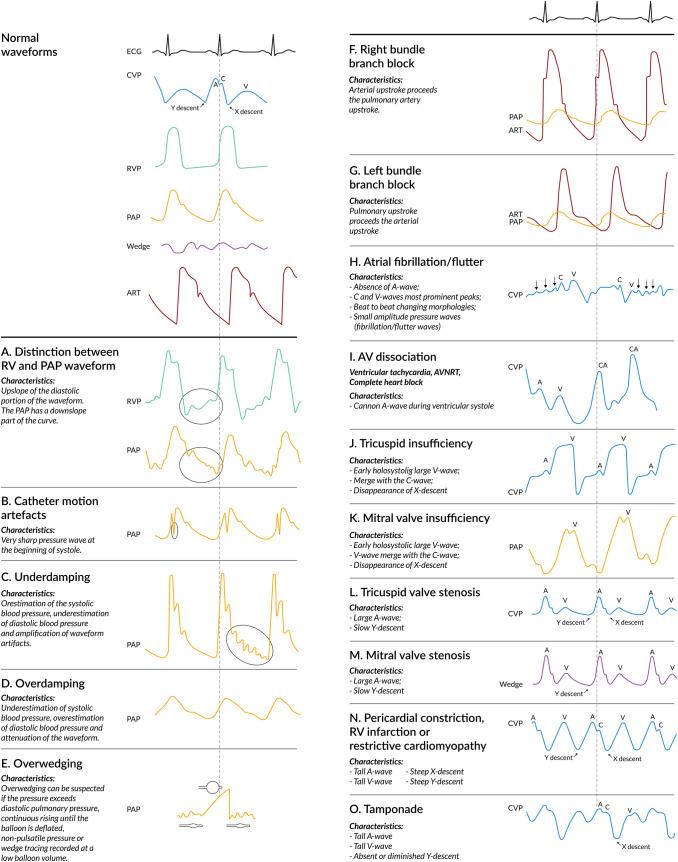
Pressure waveform pitfalls and abnormalities . *CA *cannon a-wave,* CVP *central venous pressure,* ECG *electrocardiogram,* RV *right ventricle*, RVP *right ventricular pressure,* PAP *pulmonary artery pressure,* ART *arterial

By advancing the catheter further with the use of an inflated balloon, the PAC will float across the pulmonary valve into the pulmonary artery, displaying a PAP waveform. The most distinctive feature in comparison to the RV pressure waveform is the increment in diastolic pressure in the pulmonary artery compared to the diastolic pressure in the normal RV (Fig. 2a). This is otherwise known as the diastolic pressure step up [23]. It is of note that this diastolic pressure step up can be minimal in the setting of right heart failure. The PAP waveform consists of 4 phases, the first being a steep, rapid systolic upstroke, which is followed by a systolic peak. In a normal PAP waveform, there should be no significant pressure difference between the peak systolic RV pressure and peak systolic PAP. The normal gradient between systolic RV and PAP is 0 to 3 mmHg [36, 37]. The third phase is the dicrotic notch, which represents the closure of the pulmonary valve, and thus the beginning of the diastole. The dicrotic notch always follows the T-wave of the ECG. After the dicrotic notch comes the diastolic run-off, which marks the diastolic phase of the waveform. Normal systolic PAP ranges between 14 and 28 mmHg, normal diastolic PAP ranges between 5 and 16 mmHg, and normal mean PAP between 10 and 22 mmHg (Fig. 1).

#### Wedge position

After further insertion the PAC will finally reach its wedge position. Balloon occlusion stops all distal flow and creates a static fluid column between the tip of the catheter and the junction point of the pulmonary veins and left atrium. The pulmonary artery wedge pressure (PAWP) is believed to reflect both the pressures in the pulmonary veins as well as in the left atrium [38]. In general, PAWP and pulmonary artery occlusion pressure (PAOP) can be used interchangeably and both refer to the same measurement. The PAWP waveform usually depicts two pressure peaks: the a-wave and the v-wave, as well as two descents called x and y. The v-wave is generally the most prominent peak. The c-wave is often difficult to discern in a normal wedge pressure trace due to the delayed representation of the left atrial pressure, the damped reflection, and a shorter time interval between atrial and ventricular contraction of the left atrium compared to that of the right atrium [39]. It is important to keep in mind that the PAWP is a delayed representation of the left atrial pressure since the pulmonary vascular bed is positioned between the PAC and the left atrium. In addition, PAWP is also a damped reflection of phasic atrial pressure waves. The amount of damping is variable; however pressure peaks can potentially be significantly underestimated [40]. As a result of this time lag, the a-wave of the wedge pressure will be visualized shortly after the R-wave on the ECG, although the a-wave represents the end-diastolic phase [39]. Since the wedge position of the balloon does not stop flow in the antegrade direction completely, PAWP is always lower than the mean PAP. After reaching the wedge position the balloon should be deflated and not advanced any further. After deflating the balloon, the PAP waveform should re-appear. If not, the catheter should be retracted for about 2 cm. PAWP should be measured at the end of the a-wave or before the QRS complex, at the end of the expiration, when pleural pressures are minimal, and should ideally be recorded as the mean of three measurements. However, most devices provide digitized mean PAWP. A normal PAWP range is between 5 and 12 mmHg (Fig. 1) [41].

### Interaction with waveforms

#### Catheter position

**Fig. 3:**
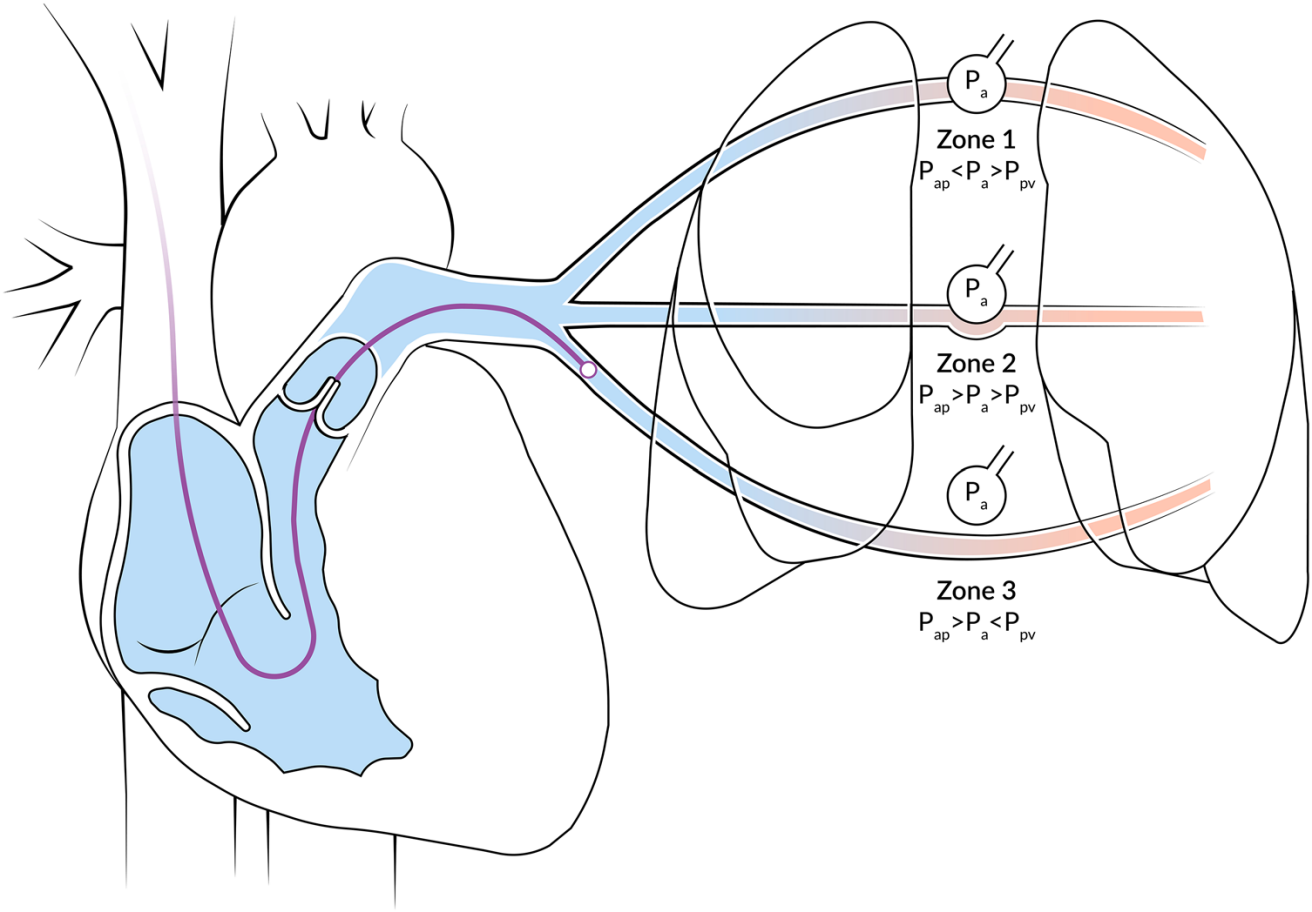
Pulmonary artery catheter location in relationship to West`s zones of the lung *P*_*ap*_ pulmonary arterial pressure, *P*_*a*_ pressure in the alveoli, *P*_*pv*_ pulmonary venous pressure

In the human lung there are 3 vertical zones, called the West zones, each with a different physiology. In West zone 1 (apex), alveolar pressure exceeds the pulmonary artery and pulmonary venous pressures. In West zone 2 (central), the alveolar pressure exceeds only the pulmonary venous pressure, and in West zone 3 (base) the alveolar pressure is lower than both the arterial and venous pulmonary pressures (Fig. 3) [42]. When the alveolar pressure exceeds the pulmonary vein pressure in West zone 1 or 2, the pressure derived at the tip of the PAC is the alveolar pressure instead of pulmonary venous pressure (or left atrial pressure; LAP or left ventricular end-diastolic pressure; LVEDP). Therefore, positioning the tip of the PAC and measurement in West zone 3 is a prerequisite for PAWP to accurately reflect LAP (Fig. 3). Absent a and v-waves, marked as PAWP variation during the respiration cycle, and pulmonary artery diastolic pressure exceeding wedge pressure (in the absence of tall a or v-waves) can indicate an incorrect wedge position in West zone 1 or 2 [43].

#### Respiratory cycle

CVP, PAP, and PAWP values should be measured at the end of expiration. At this point, the pleural pressure is closest to atmospheric pressure, and thus the influence of pleural pressures on measurements which are being compared to atmospheric pressure is minimal, both during spontaneous and positive pressure ventilation. Exceptions from this rule are spontaneously breathing COPD patients with forced expiration, where CVP should be measured early in expiration, before the patient begins to push. One should be aware of the fact that this may not necessarily be the highest or lowest pressure measured during the respiratory cycle [44].

#### Airway pressure

Positive end-expiratory pressure (PEEP), either intrinsic or extrinsic, can influence measured pressures by its effect on pericardial pressure. During spontaneous breathing, and even during positive pressure ventilation with zero end-expiratory pressure, the pericardial pressure is minimal at the end of expiration. With PEEP applied, the pericardial pressure exceeds zero and can lead to overestimation of LVEDP and CVP [33, 38]. Therefore, CVP and PAWP might not be true indications of LAP when a patient is receiving a PEEP of 10 cmH_2_0 or more [45]. Different methods to correct for applied PEEP are suggested, including various formulas or abrupt airway disconnection [45–48]. An often used formula is: corrected pressure (mmHg) = measured pressure (mmHg) – [0.5x (PEEP/1.36)] [49]. Awareness of possible overestimation of PAWP due to PEEP from various lung compliance during mechanical ventilation is critical in the correct interpretation of the data.

### Waveform pitfalls

The most common artefact is the catheter whip [50]. At the onset of systole, the catheter may be set into motion by closure of the tricuspid valve and by RV contraction. Fluid within the catheter might accelerate due to the movement of the catheter, or the catheter might strike either the walls of the heart or pulmonary artery. In the waveform, a very sharp pressure wave will appear at the beginning of systole (just after the R-wave of the ECG) and will only be visible in the RV and PAP tracing (Fig. 2b). This will not only result in a waveform artefact but also in artefactual pressure peaks [51]. Repositioning of the catheter with 1 or 2 centimetres may be helpful when trying to obtain more accurate pressures. It is of note that, although it might result in more accurate pressures, the artefact will probably still be visible.

#### Damping

Catheter-transducer monitoring systems have three characteristic physical properties: elasticity, mass, and friction. These properties determine the system’s operating characteristics, referred to as the dynamic response. An optimal dynamic response is required to measure pressures accurately. The dynamic response is characterized by both the natural frequency and the damping coefficient. The natural frequency describes how rapidly the system oscillates and the damping coefficient describes how rapidly it comes to rest [52]. The fast-flush test has been invented in order to evaluate the dynamic bedside response by briefly giving a fast flush several times, preferably during the diastolic pressure run-off [53]. The clinician should observe the natural frequency by counting the distance between oscillations, and the damping coefficient by counting how quickly the systems returns to baseline. In an optimally damped waveform, 1.5 to 2 oscillations are seen. When there are more oscillations, the system is underdamped; when there are less, the system is overdamped. An underdamped system will overestimate the systolic blood pressure and/or underestimate diastolic blood pressure, which will result in amplification of waveform artifacts (Fig. 2c). Overdamped systems will underestimate systolic blood pressure and/or overestimate diastolic blood pressure (Fig. 2d) [53]. Due to the intrinsic properties of the monitoring set-up, waveform analysis at high heart rates might be unreliable and difficult to execute.

Failure to remove all air from the catheter or tubing, or obstruction of the pressure channel of the catheter by blood clots, might result in overdamping of the waveform, which would lead to falsely low systolic pressure measurements. In case of an underdamped system, it is not advised to introduce a small air bubble into the tubing. By adding an air bubble the natural frequency of the system will be lower, resulting in further amplifying systolic pressure overshoot [53]. A clinician should be aware of artefacts producing erroneous values on the monitor. This can be the result of artefactual pressure troughs, resulting in nadir pressures that are recorded as diastolic pressures but which are not the factual diastolic pressure. Advancing or withdrawing the catheter might be helpful when removing the artefact and replace the pressure with a more accurate measurement of the diastolic pulmonary artery pressure [52]. In the event that overdamping or underdamping cannot be corrected, the clinician may consider replacing the catheter. If this is impossible or undesirable, the clinician should not use the absolute values of systolic and/or diastolic PAP for the correct interpretation of the clinical situation. However, the trend of these variables over time may still reflect actual hemodynamic changes.

#### Elevated right ventricular pressures

In case end-diastolic pressure of the RV is elevated, as in RV failure, it might be difficult to distinguish RV pressure from PAP. Close examination of the diastolic component of the waveform is likely to reveal the answer, since a diastolic step up is limited in the setting of right heart failure. The PAP is always going to decrease during the diastolic phase (after the dicrotic notch), as blood flows toward the left atrium, whereas the pressure in the RV steadily increases during diastole due to filling of the RV. In addition, the RV waveform can also depict a notch, called the incisura, caused by closure of the pulmonic valve. However, this notch will originate simultaneously with the T-wave instead of after the T-wave, as is the case with the dicrotic notch of the PAP waveform (Fig. 2a) [23]. Analysis of the RV waveform can be useful in early detection and subsequent management of RV dysfunction, especially during cardiac surgery [35, 54]. Under conditions of impaired RV function, the diastolic slope may change. In the early stage of RV failure, the diastolic phase is characterized by a progressively oblique upslope. During severe impaired RV function the diastolic RV waveform will become square-root shaped. In addition, elevated systolic pressures have been described in the setting of RV outflow tract obstruction. This condition, defined as a pressure gradient between RV and PAP of at least 25 mmHg, can happen in up to 4% of cardiac surgery patients and is associated with hemodynamic instability [37]. Since RV pressure monitoring requires a different PAC with a dedicated RV pace-port, further details are beyond the scope of this review. An excellent review of this topic is provided by Raymond and colleagues [35]. It is of note that the PAC used for RV pressure monitoring does not enable continuous cardiac output and RV ejection fraction measurements.

#### Overwedging and underwedging

Overwedging occurs when eccentric balloon inflation causes the catheter tip to occlude against the pulmonary artery wall, after which it thus no longer measures intravascular pressure. Instead, pressure is now produced by a pressurized continuous flush system as it builds up against obstructed distal opening. Overwedging can be suspected under any of the following circumstances: if the pressure exceeds diastolic pulmonary pressure, if the waveform continuously rises until the balloon is deflated, if the pressure is non-pulsatile, and/or if wedge tracing is recorded at a low balloon volume (< 1.5 cc) (Fig. 2e). Since overwedging is mostly caused by distal migration of the catheter, the solution is usually to withdraw the PAC to a more proximal position [51]. It is of note that inflation of the balloon, while the catheter is migrated to a distal position, should be avoided because it may cause rupture of a small pulmonary vessel, which can lead to serious lung hemorrhage. In patients with high PAP, underwedging can occur from incomplete occlusion of the pulmonary artery branch, which is related to poor compliance of the pulmonary arteries and will lead to an overestimation of the PAWP [55].

### Waveform abnormalities

Several clinical pathologies can have impact on PA waveform appearances. All described clinical conditions and their corresponding waveforms can be found in Fig. 2.

#### Heart rhythms and bundle branch blocks

When interpreting waveforms, simultaneous observation of pulmonary artery waveforms with the ECG registration and with arterial waveform monitoring could be useful. Under normal conditions, the PAP upstroke precedes the arterial upstroke due to the longer duration of left ventricular isovolumetric contraction [56]. Since this lag time is small under normal conditions, the waveforms may seem to overlap. However, the presence of a bundle branch block may alter this relation between PAP and systemic arterial pressure. A left bundle branch block delays left ventricular contraction, increasing the lag time between the PAP upstroke and arterial upstroke even more (Fig. 2g). A right bundle branch block has the opposite effect; arterial upstroke now precedes PAP upstroke (Fig. 2f) [23]. Tachycardia might produce fusion of waveform components, particularly the a and c-waves, whereas bradycardia can reveal a mid-diastolic plateaus pressure wave (h) between the x-descent and v-peak [57]. In case of atrial fibrillation, the a-wave will disappear from the CVP waveform due to the loss of atrial contraction. The c-wave is more prominent compared to normal sinus rhythm due to high end-diastolic atrial volume and subsequent isovolumetric ventricular contraction, displacing the tricuspid valve toward the right atrium. Atrial fibrillation leads to variability in chamber filling, and thereby to the contractile state with concurrent changes in waveform morphologies. In addition to the c- and v-waves, small amplitude pressure waves may be superimposed to the waveform, reflecting atrial activity (Fig. 2h) [57, 58]. In case of atrioventricular dissociation (ventricular tachycardia, complete heart block, re-entry tachycardia), cannon a-waves are inscribed in the CVP waveform because of atrial contraction against a closed tricuspid valve during systole. Cannon a-waves may occur before, during, or after the c-wave. Cannon a-waves can also be noted in the wedge pressure waveform (Fig. 2i) [59].

#### Tricuspid valve disease

In case of severe tricuspid regurgitation, blood leaks back from the RV towards the right atrium across the incompetent valve. This will result in an early systolic large v-wave on the CVP waveform. Since this v-wave is holosystolic, it will merge with the c-wave and make the x-descent disappear (Fig. 2j) [60]. Tricuspid stenosis causes an obstruction between the right atrium and the RV, resulting in diminished right atrial emptying, impaired RV filling, and elevation of mean CVP. Tricuspid stenosis affects the diastolic portion of the CVP; the waveform will depict a prominent a-wave and a slow y-descent (Fig. 2l). Other diseases which impair RV filling by increasing RV stiffness (RV infarction, pericardial constriction, pulmonic stenosis, pulmonary hypertension) may produce a prominent end-diastolic a-wave and a taller v-wave, but the y-descent should be preserved [57, 58].

#### Mitral valve disease

Mitral valve regurgitation has similar implications for the PAP/PAWP waveform as the previously described tricuspid regurgitation has for the CVP waveform. The holosystolic prominent v-wave with fusion of the c-wave and obliteration of the x-descent will define the PAP and PAWP waveform in the presence of mitral valve regurgitation (Fig. 2k). However, due to the delayed, damped reflection of the left atrial pressure, c-wave merging can be less evident [60]. It is of note that the height of the v-waves does not predict the intensity of the mitral valve regurgitation [61]. The presence of a large v-wave in PAWP waveforms may complicate a true distinction between PAWP and PAP waveform. In case this happens, drawing a comparison with the ECG and arterial waveform may be helpful. The PAWP will start after both the arterial upstroke and the T-wave on the ECG, while the PAP will slightly precede both systemic arterial pressure upstroke and the T-wave [62]. Like tricuspid stenosis in the CVP waveform, the PAWP waveform will depict a prominent end-diastolic a-wave, and a slow y-descent in case of mitral valve stenosis (Fig. 2m). Increased LV stiffness (left ventricular infarction and hypertrophy, pericardial constriction, aortic stenosis, and systemic arterial hypertension) will produce a prominent a-wave, but the y-descent should be preserved [60].

#### Restrictive physiology

In pericardial constriction, the PAP waveform is markedly different. All of the waveform components are amplified; tall a and v-waves with steep x and y-descents are visible, creating a sawtooth M (in case of a fast heart rate) or W configuration (in case of a slow heart rate) [58]. These morphologic features may also be seen in the CVP waveform of patients with RV infarction or restrictive cardiomyopathy, since both pathologic conditions share the same underlying pathophysiologic mechanisms (Fig. 2n) [63, 64].

#### Cardiac tamponade

Compression of the heart due to pericardial fluid results in an increased CVP, as well as in a reduced cardiac diastolic volume, stroke volume, and cardiac output. Despite the hemodynamic similarities between pericardial constriction and tamponade, the PAP waveform is slightly different [65]. The characteristic of the CVP waveform in cardiac tamponade is monophasic and dominated by a systolic x-descent. The y-descent is diminished, or altogether absent, due to impaired RV filling (Fig. 2o) [58]. This is caused by the difference in blood flow from the vena cava to the right atrium between pericardial constriction and tamponade. In cardiac tamponade, venous return to the right atrium is limited to the period of atrial relaxation (x-descent), whereas in restrictive pathophysiology, it is biphasic with a peak during atrial relaxation and early ventricular filling (x- and y-descent) [65].

#### Left ventricular end diastolic pressure

According to the principle of communicating tubes, the PAWP may be used as an indicator of LV filling pressure (LVEDP). The mitral valve is open at the end of diastole and thus, to some extent, PAWP represents the pressure in the left atrium and LV as well. However, these pressures are not necessarily the same. The LVEDP determines the force of ventricular contraction, whereas the mean left atrial pressure is the pressure level which, on average, must be exceeded if blood is to return to the heart [66]. The true filling pressure is the net result of the intracavitary LVEDP and the transmural pressure. Therefore, pericardial pressures (or juxtacardiac pressures) and mediastinal pressures should be taken into account. Under normal conditions, these pressures are respectively zero and between − 1 and − 3 mmHg, and thus PAWP is assumed to accurately reflect LVEDP [67]. However, in certain specific pathophysiological situations, measurements of PAWP do not accurately reflect LVEDP due to changes in pericardial or mediastinal pressures. Underestimation can occur during diminished LV compliance, in case of obstruction of pulmonary blood flow, during aortic or pulmonic valve regurgitation, or during right bundle branch block. Overestimation can be caused by positive end-expiratory pressure, pulmonary veno-occlusive disease, pulmonary arterial hypertension, mitral valve stenosis or regurgitation, tachycardia, or ventricular septal defect [43].

## Conclusions

Adequate catheter placement and detailed understanding of PAC-derived waveform characteristics are a prerequisite for the correct interpretation of physiology and pivotal in clinical decision making. In the second part of this review, we will describe technical features, clinical applications, limitations, and complications of the contemporary PAC.

## Abbreviations

CCO: Continuous cardiac output
COPD: Chronic obstructive pulmonary disease
CVP: Central venous pressure
ECG: Electrocardiogram
EDV: End-diastolic volume
ICU: Intensive care unit
IJV: Internal jugular vein
LAP: Left atrial pressure
LV: Left ventricle
LVEDP: Left ventricular end-diastolic pressure
PA: Pulmonary arterial
PAC: Pulmonary artery catheter
PAOP: Pulmonary artery occlusion pressure
PAP: Pulmonary arterial pressure
PAWP: Pulmonary artery wedge pressure
PEEP: Positive end-expiratory pressure
RCT: Randomized controlled trial
RV: Right ventricle
ScvO_2_: Central venous oxygen saturation
SvO_2_: Mixed venous oxygen saturation
